# The Obligations of the World to Hahnemann

**Published:** 1882-05

**Authors:** Edward Bayard

**Affiliations:** New York


### HOMŒOPATHIC PHYSICIAN.

A MONTHLY JOURNAL OF MEDICAL SCIENCE.

"If our school ever gives up the strict inductive method of Hahnemann, we are lost, and deserve only to be mentioned as a caricature, in the history of medicine."—CONSTANTINE HERING.

Vol. II. MAY, 1882. No. 5.

### THE OBLIGATIONS OF THE WORLD TO HAHNEMANN.

EDWARD BAYARD, M. D., NEW YORK.

Among human minds two classes are pre-eminent. First, those which most consider effects, phenomena. Second, those which most consider the underlying principles by whose action effects and phenomena are developed. Both these classes are useful, and help one another. But the great minds which stand out as beacon lights along the pathway of human progress, belong either to the second class, which gives most attention to the eternal principles of things, or else to a third and still higher class, to wit: those which combine the best elements of the other two, and excel both in scientific observation of facts, and the philosophical generalization upon facts which have been established.

We speak of underlying principles—of eternal principles. What do we mean by these terms? What is the thing we call a principle?

A principle of nature is simply a mode of divine action—or the way in which God does something. For example, what is an astronomical principle? It is simply the way in which God maintains the harmonious movement of the celestial bodies. You may call it centripetal force, if you please—you may call it centrifugal force—you may call it the law of gravitation—you may call it anything—but it is simply a mode of divine action, a way in which the power of God is exerted to produce results. It is the same in every field of science—in chemistry, in medicine and biology. Analysis always carries us back to a mode in which God operates, and when we strike that we call it a principle—an underlying principle, an eternal principle.

The great benefactors of the human race have been those who have discovered these principles, and so set them forth, that others could go on with the work of discovery and application until the most beneficial results were obtained.

We should not lose sight, however, of the scientific observers of facts, whose dry labors and inconspicuous drudging often furnish the material with which great philosophical minds elaborate principles. Tycho Brahe patiently recorded his astronomical observations for years without perceiving their significance. Kepler did perceive their significance, and from them his vast genius and patient research evolved his three great laws of planetary motion.

Our Hahnemann was a Tycho Brahe and a Kepler in one. He made his own observations, tabulated his own facts, and then evolved from them the laws or principles which originated and governed the facts. His everlasting credit is that he did this so well, and made it so plain, that his disciple can go on forever, extending the principles revealed by their master. He opened a fountain of healing that shall never be closed.

On this, the 10th day of April, more than a century ago, was born SAMUEL HAHNEMANN. He was created for the great work he was destined to fulfill. His mind was organized for it. No man since the days of Aristotle united in one person such power of both observation and generalization. His mind was so logical that it never permitted him to wander into hypothesis. His observation was peculiarly exact. He had a conscience alive to every sense of duty in the fulfillment of his mission. His heart was in sympathy with the suffering of humanity in its struggle with the visitation of disease, and held him to his work through ridicule, misrepresentation and obloquy. As the moon passing through clouds which threaten to obscure her brightness, at last breaks into acknowledged light, seen of all men, so SAMUEL HAHNEMANN, with patient virtue, passing through evil and good report, held his way.

What are the obligations of the world to SAMUEL HAHNEMANN?

He brought to light the law that drugs produce in their action a specific moral state, altering the natural state of feeling and thought. This knowledge is of the utmost interest to humanity. For if man has an individualism intended by his Maker to work out his usefulness and his destiny, a work reaching to ages to come—and if a drug may break up that *proprium*—that individuality—it is something of the utmost consequence for man to know, let alone its value in the selection of remedies. Permit me to illustrate from experience. I was called to see a lady, supposed by her friends to be insane on account of the wonderful change in her character. Genial in her nature, social and sympathetic in her disposition, she gathered many friends around her. A change took place in her disposition; she became hypochondriacal in humor, taciturn, repugnant to conversation, abstracted. Her friends supposed, not without reason, that she was becoming deranged. In this state I found her. I saw upon a table some dark-colored liquid in a bottle. I asked what it was. She said that it was a tonic that had been recommended. It was *Chamomilla*, and she was under its moral action. I refer you to the moral states produced by *Chamomilla vulgaris* in the provings of HAHNEMANN. This so-called tonic had changed her nature so that all thought she was mentally deranged. The antidotes to *Chamomilla* gradually restored her to her natural state.

What a serious influence drugs must exert upon the expanding and sensitive brain of a young child, when, perhaps, a lasting impression is made upon the nervous centre, and the future character and disposition of a man or woman are shaped and directed by a drug! Surely the world is under obligation to SAMUEL HAHNEMANN. For the cure of the insane, what an essential knowledge has he given to the homœopathic physician! Without it he could not succeed in the cure of mental disease. And I have no doubt that the use and abuse of drugs have been the direct producing cause of many cases of insanity, heretofore supposed to arise from an unaccountable obliquity of mind and feeling. Surely the world is under obligation to SAMUEL HAHNEMANN for the discovery of this law.

He discovered, too, the nature and effect of chronic disease, arising from hereditary taint or direct inception, to hold back and delay the curative action of remedies which would otherwise have ended quickly in perfect equalization of the vital forces. This chronic disease, having no locality, may, by the irritation of the new disturbance, act with it, giving an intensified constitutional state. This is seen strikingly in intermittent fever, which, by the chronic disease mingling with the impression of malaria, is made obstinate. The perturbation excited by malaria rouses the chronic miasm, and gives it a constitutional character and delays the reactive process. Our homœopathic brethren who are ignorant of this law, or overlook it, give for intermittent fever massive doses of *Quinine*, declaring, to the great discredit of the Hahnemannian system, that intermittent fever cannot be cured without doses of *Quinine*, and by their revulsive treatment, breaking up and shattering the constitution—*the constitution* which is the power of reaction impressed upon the vital force by an all-wise God. Surely the world is under obligation to SAMUEL HAHNEMANN for the knowledge of this law.

The greatest of the obligations of the world to HAHNEMANN is for the true law of cure. He not only discovered the facts in which the law rested, but was the sole discoverer of the principle which governed them all. That great law was, that "like cures like"—not the same. If any cures are wrought in this mortal frame of ours, it is the work of nature. Whenever the vital forces are disturbed by a morbific cause, there is inherent in nature an established law of the vital force, which is a tendency to react against the disturbing cause until an equilibrium of that force is established. That equilibrium is health. And if that tendency to reaction is overwhelmed or delayed, a similar irritant like unto the diseased movement being given, arouses that tendency of nature with increasing power to overcome the disease—which is what nature is striving to do, and what she, in the end, will accomplish if she is not enfeebled by the disturbing cause. This is the homœopathic law and its operation. He that would succeed in our profession must apply a remedy closely, and within conservative limits, and *look to the reaction alone.* We ignore the allopathic art with its narcotics and palliatives, and disregard of the law of nature, and those who attempt to generalize hypothetically on pathological states, and on that hypothesis prescribe massive doses that produce alterative effects, and in their alteration break down the resisting power of the system.

I once knew a physician of high standing in the allopathic school, who after he had made himself master of the materia medica of HAHNEMANN and the law of its application, said: "I have killed many a man and never knew how till now." He innocently gave, not knowing the specific action of the drug, massive doses, which, falling with crushing power along the line of the weakness of his patient, prevented nature's reaction. I knew a young man, brought up in the country, with a strong constitution, suffering under mumps, to die from one dose administered by an allopathic physician, of six grains of *Mercury*. He died in three days from the intense action produced by a similar irritant, which, if it had been administered in smaller doses of the same medicine, would have restored him to health. Nature was outraged—overwhelmed. The constitution was broken down. Is not the world under obligations to SAMUEL HAHNEMANN?

May it not be truly said of HAHNEMANN; no more worshipful figure ever stood in the forefront of a nation's life than that of this physician who broke free from the bondage of the whole world's professional traditions and beliefs, and patiently gave his life to the discovery of the true law of cure and the first proving of remedies to cure every disease that can afflict the human body?

